# Trombose Mecânica da Válvula Mitral em Paciente com Infecção por COVID-19

**DOI:** 10.36660/abc.20210581

**Published:** 2022-06-06

**Authors:** Emre Aruğaslan, Yunus Çalapkulu, Ender Örnek, Mustafa Karanfil, Hüseyin Bayram, Seref Alp Küçüker

**Affiliations:** 1 Hospital da Cidade de Ancara Departamento de Cardiologia Ancara Turquia Departamento de Cardiologia, Hospital da Cidade de Ancara, Ancara – Turquia; 2 Hospital da Cidade de Ancara Departamento de Cirurgia Cardiovascular Ancara Turquia Departamento de Cirurgia Cardiovascular, Hospital da Cidade de Ancara, Ancara – Turquia

**Keywords:** COVID-19/complicações, Valva Mitral/cirurgia, Inflamação, Trombose, Transtornos da Coagulação Sanguínea/complicações

## Introdução

A nova doença de coronavírus-2019 (COVID-19) causada pelo “*Severe Acute Respiratory Syndrome Coronavirus-2*” (SARS-CoV-2) tornou-se uma pandemia global. Embora o envolvimento respiratório seja a apFontes de financiamentoresentação predominante, as evidências atuais mostram que a COVID-19 é uma doença multissistêmica com coagulopatia e complicações tromboembólicas. O aumento da produção de fator tecidual e a redução da fibrinólise da trombina devido à hiperinflamação são os mecanismos propostos da trombose induzida por COVID-19.^1^

Apresentamos um caso de um paciente infectado por COVID-19 com trombose da válvula mitral mecânica.

## Relato de caso

**Figura 1:**
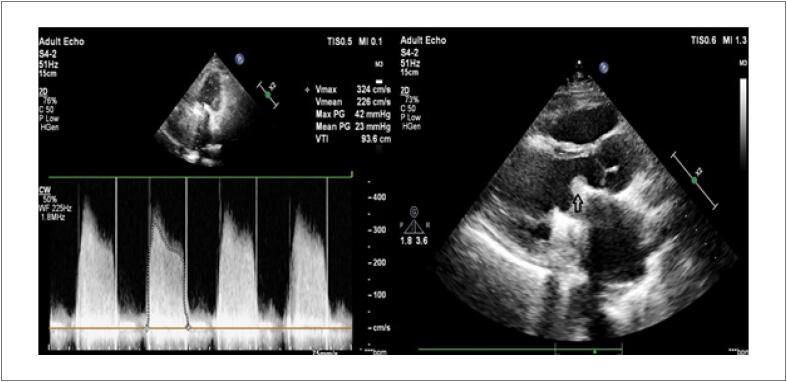
Imagens ecocardiográficas de válvula mecânica obstruída.

Paciente do sexo masculino, 46 anos, submetido à troca valvar mecânica mitral há 3 anos, foi admitido com história de 1 semana de dispneia leve e mal-estar. O exame físico revelou ausência de clique protético. Não havia distensão venosa jugular nem estertores à ausculta pulmonar. O paciente estava hemodinamicamente estável. O eletrocardiograma mostrou ritmo sinusal com alterações inespecíficas do segmento ST. As medicações regulares consistiam apenas em varfarina 5 mg/dia. Seu histórico médico recente foi notável devido à infecção por COVID-19 na sua casa. Decidiu-se testar o paciente para COVID-19 devido ao contato próximo e febre subfebril (37,5°C). O teste de swab nasofaríngeo de reação em cadeia da polimerase em tempo real foi positivo para SARS-CoV2. A tomografia computadorizada de tórax realizada no pronto-socorro revelou infiltrações centrolobulares bilaterais, que foram relatadas como atípicas de COVID-19. O ecocardiograma transtorácico (ETT) detectou mobilidade dos folhetos severamente restrita, com gradiente transvalvar médio de 23 mmHg (Figura 1). Foi observado trombo obstrutivo com diâmetro de 2,2 x 0,8 cm estendendo-se até a via de saída do ventrículo esquerdo (Figura 1, Vídeo 1). A fluoroscopia também mostrou mobilidade restrita dos folhetos. O INR de admissão foi de 3,26. Os prontuários médicos revelaram medições mensais do INR terapêutico antes da internação. Não houve outro episódio trombótico na história médica pregressa do paciente. Havia hipoxemia leve (PaO2:71 mmHg) na gasometria arterial. Os exames laboratoriais iniciais mostraram níveis de dímero D 1,0 mg/L (< 0,55), proteína C-reativa 0,02708 g/L (0 - 0,005), IL-6 14,7 pg/mL (0 - 3,4), plaquetas 258 x 10^9 /L(150-400), e ferritina 58 µg/L (22 – 322). Hemoculturas foram obtidas para descartar endocardite infecciosa. A cirurgia de emergência foi recusada devido à estabilidade hemodinâmica e infecção ativa por COVID-19.

O paciente foi internado na unidade de terapia intensiva para monitorar sintomas e hemodinâmica. A varfarina foi interrompida e a heparina não fracionada intravenosa foi administrada com dosagem guiada de aPTT. O paciente foi monitorado de perto quanto a sinais de insuficiência cardíaca e instabilidade hemodinâmica. No terceiro dia de tratamento, o ETT mostrou gradientes valvares mitrais diminuídos (média de 12 mmHg). O tratamento com heparina foi continuado. No entanto, o paciente piorou por causa de taquicardia supraventricular e edema pulmonar subsequente no dia 7. O ecocardiograma à beira do leito foi realizado imediatamente e demonstrou reelevação do gradiente pressórico médio para 28 mmHg. Foi administrado trombolítico emergente em bolus de 10 mg de tPA e infusão de 90 mg em 90 minutos; entretanto, nenhuma melhora foi observada nos parâmetros clínicos e ecocardiográficos após a lítica. Foi necessária a substituição urgente da válvula mitral. As aderências de cirurgia cardíaca prévia foram liberadas após refazer esternotomia mediana. A circulação extracorpórea foi estabelecida com canulação venosa. Trombose foi observada na valva mecânica pela abordagem da atriotomia esquerda. A válvula mecânica trombosada foi excisada e uma nova válvula mecânica (29 mm, Sorin) foi substituída. Recebeu alta com um INR alvo de 3,5 após cuidados pós-operatórios sem intercorrências. Como a infecção por COVID-19 deveria ser o gatilho da trombose da válvula mecânica, nenhuma investigação hematológica adicional foi feita. O paciente não apresentou nenhum evento adverso após a alta.

## Discussão

Descrevemos um caso de trombose mecânica da válvula mitral em um paciente com COVID-19. As complicações trombóticas do sistema cardiovascular são evidentes na literatura. Houve relatos de casos de tromboembolismo venoso e trombose de artéria coronária relacionados ao COVID-19.^2,3^ A trombose da valva mitral bioprotética foi tratada com sucesso pelo início da anticoagulação em um paciente idoso com COVID-19.^4^ As diretrizes recomendam pelo menos dose profilática de heparina de baixo peso molecular para todos os pacientes hospitalizados com COVID-19 na ausência de contraindicações absolutas.^1^

A trombose mecânica da válvula cardíaca é uma complicação com risco de vida que requer diagnóstico e tratamento imediatos. Geralmente está associada à anticoagulação inadequada. O ETT e a ecocardiografia transesofágica (ETE) são essenciais para o diagnóstico e determinação do grau e causa da disfunção valvar. O ETE não foi realizado neste paciente com COVID-19 devido ao risco aumentado de disseminação de SARS-Cov-2. A cinefluoroscopia fornece informações adicionais sobre a mobilidade e abertura do folheto. A substituição valvar de emergência é recomendada para trombose obstrutiva de prótese valvar em pacientes críticos, mas a fibrinólise deve ser considerada se o risco cirúrgico for alto.^5^ Baixo risco de sangramento, envolvimento das valvas direitas, primeiro episódio de trombose valvar e trombo menor que 1 cm² são outros fatores que tornam a fibrinólise mais favorável.^6^ A equipe cardíaca decidiu administrar fibrinolítico devido a preocupações com hiperinflamação perioperatória e hipercoagulabilidade associada ao COVID-19,^7^ mas a reoperação acabou sendo necessária após a falha dos trombolíticos.

A infecção por COVID-19 tem sido associada ao aumento da mortalidade em pacientes submetidos à cirurgia cardíaca.^8^ A resposta inflamatória exagerada ao vírus pode aumentar o risco de síndrome do desconforto respiratório agudo (SDRA) no pós-operatório.^9^ Um caso de trombose pós-operatória aguda da válvula aórtica e subsequente embolia coronária foi relatada.^10^ O risco de transmissão perioperatória do vírus para os profissionais de saúde também deve ser considerado. Entretanto, retardar a cirurgia em um paciente com trombose de prótese valvar também é arriscado devido a complicações como choque cardiogênico, insuficiência cardíaca e embolia sistêmica. A decisão entre cirurgia e trombólise para trombose valvar mecânica deve ser individualizada. Fatores clínicos, experiência local e experiência cirúrgica são fatores críticos na via de decisão.

## Conclusões

A literatura tem dados consistentes sobre a hipercoagulabilidade na infecção por COVID-19, então presumimos que a doença por Coronavírus foi o fator predisponente no desenvolvimento de trombose de válvula mecânica em um paciente com valores terapêuticos de INR. No entanto, deve-se notar que a trombose se desenvolveu, embora os marcadores pró-inflamatórios estivessem moderadamente elevados. Da mesma forma, foi relatada trombose coronariana recorrente em um caso moderado de COVID-19,^2^ portanto, a hiperinflamação pode não ser a única via que leva à trombose em pacientes com COVID-19.

Os médicos devem estar cientes das complicações trombóticas durante este surto. O uso preventivo e terapêutico de drogas antitrombóticas deve ser feito em paralelo às recomendações formais para mitigar a carga trombótica em pacientes com COVID-19.^1^

## *Material suplementar

Para assistir ao vídeo suplementar, por favor, clique aqui.
