# Supplementary material for: Trombose Mecânica da Válvula Mitral em Paciente com Infecção por COVID-19
Source: Arq Bras Cardiol. 2022 Jun 6;118(6):1141–3. [Article in Portuguese] doi: 10.36660/abc.20210581 (PMC9345140; doi:10.36660/abc.20210581)
Supplement: Translation

# Translation: Mechanical Mitral Valve Thrombosis in a Patient with COVID-19 Infection

**Authors:** Emre Aruğaslan, Yunus Çalapkulu, Ender Örnek, Mustafa Karanfil, Hüseyin Bayram, Seref Alp Küçüker

**Affiliations:** 1 Ancara Turkey Departamento de Cardiologia, Hospital da Cidade de Ancara, Ancara – Turkey; 2 Ancara Turkey Departamento de Cirurgia Cardiovascular, Hospital da Cidade de Ancara, Ancara – Turkey

**Keywords:** COVID-19/complications, Mitrakl Valvesurgery, Infammation, Thrombosis, Blood Coagulation, Disorders/complications

## Introduction

The novel coronavirus disease-2019 (COVID-19) caused by “Severe Acute Respiratory Syndrome Coronavirus-2” (SARS-CoV-2) became a global pandemic. Although respiratory involvement is the predominant presentation, current evidence has shown that COVID-19 is a multisystemic disease with coagulopathy and thromboembolic complications. Increased production of tissue factor and thrombin reduced fibrinolysis due to hyperinflammation are the proposed mechanisms of COVID-19 induced thrombosis.^1^

We present a case of a COVID-19 infected patient with thrombosis of the mechanical mitral valve.

## Case Report

A 46-year-old male patient who underwent mitral mechanical valve replacement 3 years ago was admitted with a 1 week history of mild dyspnea and malaise. Physical examination revealed the absence of prosthetic click. There was neither jugular venous distension nor rales on lung auscultation. The patient was hemodynamically stable. Electrocardiography showed sinus rhythm with nonspecific ST-segment changes. Regular medications consisted of only warfarin 5 mg/day. His recent medical history was remarkable due to the COVID-19 infection in his household. It was decided to test the patient for COVID- 19 because of close contact and subfebrile fever (37.5 °C). Real-time polymerase chain reaction nasopharyngeal swab test was positive for SARS-CoV2. Chest computed tomography scan performed in the emergency department revealed bilateral centrilobular infiltrations, which were reported as atypical COVID-19. Transthoracic echocardiography (TTE) detected severely restricted leaflet mobility, with a mean transvalvular gradient of 23 mmHg (Figure 1). Obstructive thrombus with a 2.2 X 0.8 cm diameter extending to the left ventricular outflow tract was seen (Figure 1, Video 1). Fluoroscopy also showed restricted mobility of leaflets. Admission INR was 3.26. Medical records revealed monthly therapeutic INR measurements before hospitalization. There was no other thrombotic episode in the patient’s past medical history. There was mild hypoxemia (PaO_2_:71 mmHg) on arterial blood gas analysis. Initial laboratory tests showed levels of D dimer 1.0 mg/L (< 0.55), C- reactive protein 0.02708 g/L (0 - 0.005), IL-6 14.7 pg/mL (0 – 3.4), platelets 258 x 10^9/L(150-400), and ferritin 58 µg/L (22 – 322). Blood cultures were obtained to rule out infective endocarditis. Emergent surgery was declined due to hemodynamic stability and active COVID-19 infection.

**Figure 1:** Echocardiographic images of obstructed mechanical valve.

The patient was admitted to the intensive care unit to monitor symptoms and hemodynamics. Warfarin was stopped, and intravenous unfractionated heparin was administered with aPTT guided dosing. The patient was closely monitored for signs of heart failure and hemodynamic instability. On the third day of treatment, TTE showed decreased mitral valve gradients (mean 12 mmHg). Heparin treatment was continued. However, the patient deteriorated because of supraventricular tachycardia and subsequent pulmonary edema on day 7. Bedside echocardiography was done immediately and demonstrated re-elevation of the mean pressure gradient to 28 mmHg. Emergent thrombolytic was administered 10 mg bolus of tPA and 90 mg infusion in 90 minutes; however, no amelioration was seen in neither clinical nor echocardiographic parameters after lytics. Urgent mitral valve replacement was needed. Adherences from previous cardiac surgery were released after redo median sternotomy. Cardiopulmonary bypass was established with venous cannulation. Thrombosis was observed on the mechanical valve by the left atriotomy approach. The thrombosed mechanical valve was excised, and a new mechanical valve (29 mm, Sorin) was replaced. He was discharged with a target INR of 3.5 after uneventful postoperative care. As COVID-19 infection was supposed to be the trigger of mechanical valve thrombosis, no further hematological investigation was done. The patient has not experienced any adverse event after discharge.

## Discussion

We described a case of mechanical mitral valve thrombosis in a COVID-19 patient. Thrombotic complications of the cardiovascular system are evident in the literature. There have been reports of venous thromboembolism and coronary artery thrombosis cases related to COVID-19.^2,3^ Bioprosthetic mitral valve thrombosis was successfully treated by the initiation of anticoagulation in an elderly patient with COVID-19.^4^ Guidelines recommend at least prophylactic dose of low molecular weight heparin for all hospitalized COVID-19 patients in the absence of absolute contraindications.^1^

Mechanical heart valve thrombosis is a life-threatening complication necessitating prompt diagnosis and treatment. It is usually associated with inadequate anticoagulation. TTE and transesophageal echocardiography (TEE) are essential for diagnosis and determining the degree and cause of valve dysfunction. TEE was not performed in this COVID-19 patient due to the heightened risk for SARS-Cov-2 spread. Cinefluoroscopic provides additional information about leaflet mobility and opening. Emergency valve replacement is recommended for obstructive prosthetic valve thrombosis in critically ill patients, but fibrinolysis should be considered if the surgical risk is high.^5^ Low risk of bleeding, involvement of the right valves, first episode of valve thrombosis, and thrombus smaller than 1 cm² are other factors that make fibrinolysis more favorable.^6^ Heart team decided to administer fibrinolytic due to concerns about perioperative hyper inflammation and hypercoagulability associated with COVID-19 ^7^ but redo surgery was eventually needed after thrombolytics failed.

COVID-19 infection has been associated with increased mortality in patients undergoing cardiac surgery.^8^ Exaggerated inflammatory response to the virus may augment the risk of acute respiratory distress syndrome (ARDS) postoperatively.^9^ A case of acute postoperative thrombosis of the aortic valve and subsequent coronary embolism was reported.^10^ The risk of perioperative transmission of the virus to health care personnel should also be kept in mind. However, delaying the surgery in a patient with prosthetic valve thrombosis is also risky due to complications such as cardiogenic shock, heart failure and systemic embolism. The decision between surgery and thrombolysis for mechanical valve thrombosis should be individualized. Clinical factors, local experience and surgical expertise are critical factors in the decision pathway.

## Conclusions

Literature has consistent data regarding hypercoagulability in COVID-19 infection, so we presumed that Coronavirus disease was the predisposing factor in the development of mechanical valve thrombosis in a patient with therapeutic INR values. However, it should be noted that thrombosis developed although proinflammatory markers were moderately elevated. Similarly, recurrent coronary thrombosis in a moderate case of COVID-19 was reported,^2^ so hyper inflammation may not be the sole pathway leading to thrombosis in patients with COVID-19.

Physicians should be aware of thrombotic complications during this outbreak. Preventive and therapeutic use of antithrombotic drugs should be done in parallel to formal recommendations to mitigate the thrombotic burden in COVID-19 patients.^1^

## *Supplemental Materials

See the Supplemental Video, please click here.
